# Supplementary material for: Improving the Modeling of Extracellular Ligand Binding Pockets in RosettaGPCR for Conformational Selection
Source: Int J Mol Sci. 2023 Apr 24;24(9):7788. doi: 10.3390/ijms24097788 (PMC10178219; doi:10.3390/ijms24097788)
Supplement: Supplementary file 1 [file ijms-24-07788-s001.zip › Protocol_Capture_RosettaGPCRPocketSize.pdf]

Protocol Capture for RosettaGPCRPocketSize

All files and examples are given at:
https://github.com/FabianLiessmann/RosettaGPCRPocketSize

Rosetta Documentation main site:
https://www.rosettacommons.org/docs/latest/Home

- RosettaCM Documentation
- Hybridize mover documentation
- RosettaScripts documentation
- Constraint documentation

Notes: Paths are relative, the Rosetta software is needed and a general understanding of homology modeling. Two examples for RosettaGPCRPocketSize are provided (ADRB1_example and CXCR4_example). In each example, four directories for a step-wise approach are given, this is not necessary to run the scripts but recommended as it facilitates the execution of RosettaGPCRPocketSize and is helpful to get used to the whole pipeline. To summarize the pipeline: With this method you generate a Ballosteros-Weinstein numbering (BW) file, which correlates your residue numbers to the BW numbering. In the second step you generate receptor individual randomized constraints (two tetrahedral constraint sets), followed by executing RosettaGPCR with in Step2 generated updated RosettaCM.xml scripts. In the final step, the results are evaluated, and the best models based on total energy and pocket volume are selected. Most scripts are written as jupyter notebook (`*ipynb`). Necessary libraries are `jupyter-notebook`, `Biopython`, `pandas`, `matplotlib`, `numpy`, `scipy` and `random`. If Anaconda3 is installed, all libraries should be in the base environment, else use `conda install -c anaconda jupyter`, `conda install -c conda-forge biopython`, `conda install -c conda-forge pandas`…. To start the script in the respective conda environment with `jupyter notebook *.ipynb`. A general anaconda documentation is given here:
https://docs.anaconda.com/anaconda/install/index.html.
All needed inputs are marked with several lines of `#############` in the script.

Necessary input from user:

- RosettaGPCR preparation (setup_RosettaCM_updated.py instead of setup_RosettaCM.py – provided in necessary_input)
- Fasta sequence (either from RosettaGPCR or self-selected for your respective receptor target)

Necessary input/scripts from RosettaGPCRPocketSize

- A BW file will be generated in Step1 with BW_assignment.ipynb, assignment.list and all_gpcrdb_num.data (or can be taken from gpcrdb.com)
- selected_pairs.list with the tetrahedral distances and Make_cst_files.ipynb for generating constraint files in Step2
- Calculate_volume_and_filtering.ipynb and selected_res_vol.list for the volume

calculation and final filtering in Step4
- All jupyter notebooks are as single books and summarized in RosettaGPCRPocketSize.ipynb

Supporting_scripts:
- requirements.txt with all respective python libraries
- example_rosetta_cm.xml contains an example script
- rosetta_cm.xml is a general example with filler for constraint input
- rosetta_cm(updated_and_commented).xml shows the difference between a RosettaGPCR and RosettaGPCRPocketSize Rosetta script for homology modeling

Step 0: Prepare the input files as described in RosettaGPCR
- Prepare templates, alignment, setup for RosettaCM (see setup_RosettaCM_updated.py) and span file
- Copy the target sequence as target.fasta into "Step1_Generate_BW_assignment"

Step 1: Generate a BW file assigning the residue numbers to BW numbering
- Change into Step1_Generate_BW_assignment and copy the following files to this directory: your receptor sequence as target.fasta, all_gpcrdb_num.data, assignment.list and BW_assignment.ipynb
- The target sequence should include a `>{receptor name}` line
- assignment.list includes the UniProt and IUPHAR name of all class A GPCRs, check if your receptor name is included in the list. The second column is used for assigning the receptor (if your receptor name is not included, add a new line with `"your {receptor name} from target.fasta" > "respective IUPHAR name found in all_gpcrdb_num.data"`
- all_gpcrdb_num.data lists all human class A GPCRs with their BW numbering and respective residues. The script is optimized for human sequence but will work for other organisms as well. When this step provides errors, the BW assignment is not working or your receptor sequence is special, add your target BW from gcprdb (https://gpcrdb.org/alignment/targetselection)
- Run BW_assignment.ipynb step-wise and produce your receptor BW file

Step 2: Generate several constraint file and update the RosettaCM script
- Change into "Step2_Generate_CST" and copy the generated BW file to here
- Copy and check if selected_pairs.list is in the same directory as the BW file and Make_cst_files.ipynb. selected_pairs.list contains the distance pairs (in BW numbering) and the respective distances. Six distances equal one tetrahedron, two tetrahedrons are selected, based on these, constraints are generated

- Update the number of desired constraint files (in the example for the sake of simplicity, only 10 are generated, in reality 100-1000 are a good option). For RosettaCM several hundred to a few thousand homology models are best (when using RosettaGPCRPocketSize for generating a pocket ensemble with relax, 100-300 are a good number), change your constraint file number accordingly (constraint file number ≙ desired model number; or: x-times the constraint file number ≙ desired homology model number → `-nstruct` in flags.options defines the number of models generated for each RosettaCM run)
- Run Make_cst_file.ipynb block-wise to generate the constraint files
- In the notebook a `#Bonus`-block will prepare a copy_cst_and_xml.sh script. This script facilitates copying the generated constraint files in the next directory and also, to build the respective xml scripts for the constraint file: each constraint file is addressed by a different xml script, the `#Bonus`-block will add a bash script with `sed` to change `"input/constraint_number"` to `"input/{receptor_name}_{constraint_file_number}.cst"`, an example can be seen in the Supporting_scripts as rosetta_cm.xml
- Before running the bash script, check your rosetta_cm.xml from Step 0: Have you used the setup_RosettaCM_updated.py and does your xml script include `ConstraintSetMover` and `FastRelax`? Compare to rosetta_cm.xml in Supporting_scripts

Step 3: Run RosettaCM/RosettaGPCR accordingly

- Change in Step3_RosettaGPCR and check that you prepared and copied all needed inputs and files: Two directories `input` and `output`, in `input`:
  - threaded template pdbs
  - disulf.txt (if necessary for your target)
  - flags.options
  - {receptor_name}_{constraint_file_number}.cst (Several constraint files)
  - rosetta_cm{number_of_constraint_file}.xml (Several xml files according to the number of constraint files)
  - span.txt
  - weight files (stage1_membrane.wts, stage2_membrane.wts and stage3_rlx_membrane.wts)
- If everything is present, run Rosetta_scripts and include a `-parser:protocol rosetta_cm{number}.xml` flag as well as `@flags.options`, example: `/PATH/TO/ROSETTA/main/source/bin/rosetta_scripts.static.linuxgccrelease -parser:protocol rosetta_cm1.xml @flags.options`
- Depending on your set-up (workstation with 24 cores vs. cluster with slurm script), write a bash script that loops to run all scripts, for homology modeling several hundreds – thousands models are necessary, for relax only several hundred

Step 4: Evaluate the pdbs, their volume and score

- Change into Step4_Filtering and copy all in Step3 generated pdb files to Step4_Filtering and also, the following files: Calculate_volume_and_filtering.ipynb, the BW File (from Step 1) and selected_res_vol.list
- selected_res_vol.list contains ten residues for the volume calculation
- Run Calculate_volume_and_filtering.ipynb block-wise, here the relevant residues for volume calculation are assigned based on BW numbering, the cartesian coordinates of the C_α atoms are extracted and saved as {file_name.pdb}.xyz, based on the ten residues the volume is calculated and saved {file_name.pdb}.xyz.vol
- These files are for debugging when there is an error with the volume
- In the final step, all volumes are concatenated in one file (all_vol.list) and the score is extracted from the pdbs (score.list) (when another scoring application was used afterwards, change this respectively)
- In the end, the script will merge the score from score.list and the volume from all_vol.list, calculate the median volume, select the pdbs within a specific bandwidth of ±200A^3 and the best five models according to the score. The number of best models and the volume bandwidth can be changed

Further notes/tips:

- Check the best models in PyMOL, Chimera or any other visualization tool → are the models well made? Is the helical bundle destroyed and the pocket too big? How does the binding pocket look like – is the geometry destroyed, the pocket collapsed or enlarged?
- Check the volume of the best models and also, the median volume – is it expected (lipid GPCRs are around 2,200-2,600A^3, most GPCRs are around 2,600A^3, some with large binding pockets go up to 3,200A^3)
- Doublecheck with gpcrdb.com your BW numbering, is everything alright?
- If your receptor has a known smaller or bigger binding pocket, change the distances in selected_pairs.list. An overview with all inactive determined class A GPCRs and their respective pocket distances is given in S1_all_inactive_distances.ods. Family related or specific receptor distances can be selected
- If you are unsatisfied with the results, change the constraint parameter selection in Step2. Currently the distance is increased by 3-7% to counter the collapse during relax, the constraint weight is increased to 2-4 and the constant bonus is between -1 and -10. Increase the weight or change the distance if necessary. Compare your target with known distances from related receptors in S1

Additional Material:

- High-Resolution Comparative Modeling with RosettaCM (doi 10.1016/j.str.2013.08.005)
  - Original paper describing method
- Protocols for Molecular Modeling with Rosetta3 and RosettaScripts
  - Tutorial paper giving overview of Rosetta, primary methods, and associated tutorials
- Original RosettaGPCR paper Improving homology modeling from low-sequence identity templates in Rosetta: A case study in GPCRs (doi 10.1371/journal.pcbi.1007597)
  - Paper describing the method and input, preparing the templates
  - The full preparation is available at www.rosettagpcr.org and www.github.com/benderb1/rosettagpcr.
- MeilerLab Tutorials (comparative modeling and more) and Constraint Tutorial
