# Supplementary material for: Improving the Modeling of Extracellular Ligand Binding Pockets in RosettaGPCR for Conformational Selection
Source: Int J Mol Sci. 2023 Apr 24;24(9):7788. doi: 10.3390/ijms24097788 (PMC10178219; doi:10.3390/ijms24097788)
Supplement: Supplementary file 1 [file ijms-24-07788-s001.zip › Supplementary_RosettaPocketSize.pdf]

Improving the modeling of extracellular ligand binding pockets in RosettaGPCR for conformational selection

Fabian Liessmann [1], Georg Künze [1] and Jens Meiler [1,2,3,4,*]

1 Institute for Drug Discovery, Medical Faculty, Leipzig University, 04103 Leipzig, Germany; fabian.liessmann@medizin.uni-leipzig.de (F.L.)
2 Department of Chemistry, Vanderbilt University, Nashville, TN 37235, USA
3 Center for Structural Biology, Vanderbilt University, Nashville, TN 37235, USA
4 Center for Scalable Data Analytics and Artificial Intelligence, Leipzig University, 04105 Leipzig, Germany
* Correspondence: jens@meilerlab.org

Supplementary

Table S1. List of selected receptors and distances. The determined structure in the best resolution in inactive mode for each receptor was selected and the distances between their C_α-atoms of selected residues calculated. Receptor name and corresponding PDB ID are given. (S1_all_inactive_distance.ods)

Table S2. List of selected receptors and pocket volume. The determined structure in the best resolution in inactive mode for each receptor was selected and the volume based on ten residues were calculated. Receptor name and corresponding PDB ID are given. (S2_all_calculated_volumes.ods)

Table S3. List of selected residues defining the pocket. All selected 34 residues spanning the binding pocket of class A GPCRs are listed in GPCRdb updated BW numbering and their respective role for volume calculation and pocket modeling.

Helix	GPCRdb numbering	For volume calculation	For restraint construction
**1**	1.39.39		
	2.57x56	x	
	2.60x59		X1
**2**	2.61x60		
	2.63x62		
	2.64x63		
	2.65x64	x	X2
	3.28x28	x	
	3.29x29		
	3.32x32		
**3**	3.33x33		
	3.36x36		
	3.37x37	x	
	3.40x40		X1
**4**	4.57x57		X2
	5.38x39	x	X1
	5.39x40		
**5**	5.42x42		
	5.43x44		
	5.46x461	x	X2
	5.47x47		
	6.48x48		X2
	6.51x51	x	
**6**	6.52x52		
	6.55x55		
	6.58x58	x	
	6.59x59		
	7.32x31	x	X1

	7.35x34	
	7.36x35	
**7**	7.39x38	
	7.40x39	
	7.42x41	
	7.43x42	x

Table S4. List of benchmarked geometries, tetrahedrons, constraint parameters, and filters. The determined structure in the best resolution in inactive mode for each receptor was selected and the distances between their C_{α}-atoms of selected residues. Several benchmark-sets were constructed over the time. Receptor name and volume difference are given for the respective parameter. (S3_List_of_benchmarked_parameters.ods)

Table S5. List of benchmarked ligands for the final benchmark-set of GHSR, GNRH, OTR and Y2R. Ligands were downloaded from PubChem as all tested compounds for each receptor, respectively, and the datasets filtered for active ligands and weights between 400 and 700 Da. The final selected five ligands per receptor were randomly selected regardless of the binding pocket and final activity.

Receptor	Ligand ID	SMILES	Molecular Weight [Da]
GHSR	16040587	C[C@@H]1CN(C[C@@H](N1)C)CC2=CC=C(C=C2)C3=C(N=CC=C3)C(=O)N4CCC(CC4)NC5=CC=C(C=C5)F	501.29
GHSR	24180646	CC(C)C[C@@H](C1=C(C(=CC=C1)F)N2CCN(CC2)C(=O)[C@@H](CC3=C(C=C(C=C3)Cl)Cl)N4CCCC4=O)NCCN	591.254
GHSR	44437966	CCN(CC)CCN1C2=CC(=CC(=C2[C@@](C1=O)(C3=CC4=CC=CC=C4C=C3)O)C(F)(F)F)C#CCCC(=O)N5CCOCC5	607.266
GHSR	70681018	CC1=CC=CC=C1OC2=CC3=C(C=C2)NC(=O)CN(C3=O)[C@H](C(C)C)C(=O)NC4CCN(CC4)CC5=CC=CC=C5	554.289
GHSR	71460577	CN1CCN(CC1)CC(=O)NC2(C3=C(C(=C(C=C3NC2=O)Cl)Cl)Cl)C4=CC=C(C=C4)Cl	500.034
GNRH	10345138	CC1=C(C(=O)N(C(=O)N1CC2=C(C=CC=C2Cl)F)C[C@@H](C3=CC=CC=C3)N)C4=CC=CC=C4F	481.137
GNRH	11720984	CC(C)(C)NC(=O)NCCC1=NC2=C(N1CC3=CC=CC=C3)C=CC(=C2)S(=O)(=O)NCC4=CC(=C(C=C4)F)F	555.212
GNRH	21046762	CC1=CC2=C(C=C1CC3=CC=C(O3)C(=O)NCC4CCN(CC4)C(=N)N)C(CCC2(C)C)(C)C	464.315
GNRH	44573713	CC1=NN(C(=C1)CN2CCN(CC2)C3=CC=CC4=C3N=C(N4)C5=CC=C(C=C5)C(C)(C)C)C	442.284
GNRH	70697569	CC(C)(C)CNC(=O)NCCC1=NC2=C(N1CC3=CC=CC=C3)C=CC(=C2)S(=O)(=O)NCC4=CC=C(C=C4)F	551.237
OTR	10054193	CC(=O)CCCC(=O)NC1C(=O)N(C2=CC=CC=C2C(=N1)C3=CC=CC=C3)CC(=O)NCCC4=CC(=C(C=C4)Cl)Cl	592.164
OTR	11570523	CC(C)C[C@@H]1C(=O)N[C@@H](C(=O)N1[C@H](C2=CC=C(C=C2)N3CCC(CC3)O)C(=O)NC(C)(C)C)C4CC5=CC=CC=C5C4	574.352
OTR	11757835	CC(=O)CCCC(=O)NC1C(=O)N(C2=CC=CC=C2C(=N1)C3=CC=CC=C3)CC(=O)NCCC4=CC5=CC=CC=C5C=C4	574.258
OTR	24981103	CCN1CCN(CC1)C2=C(C=C(C=C2)S(=O)(=O)N3CCCCC3)NC(=O)CC4=CC(=C(C=C4)Cl)Cl	538.157
OTR	46233065	CC1=C(C=CC(=C1)C(=O)N2CCCNC3=CC=CC=C32)CNC(=O)N4CCC[C@H]4C(=S)N5CCCN(CC5)C	548.293
Y2R	3247171	CN(C)C(=O)C1=CC2=C(N1CC3=CC=CC=C3)C[C@@H]4[C@H]2[C@](N(C4)C(=O)C5=CC=CC=C5)(CC6=CC=C(C=C6)F)C(=O)OC	579.253
Y2R	3431315	C1CCN(CC1)S(=O)(=O)C2=CC(=C(C=C2)Cl)NC(=O)C3=CC=CC=C3NS(=O)(=O)C4=CC=C(C=C4)F	551.075
Y2R	6438437	C[C@H]\\1C/C=C/[C@H]2[C@H]3[C@](O3)([C@H]([C@@H]4[C@@]2(C(=O)/C=C/C(=O)[C@@H](/C(=C1)/C)O)C(=O)N[C@H]4CC5=CNC6=CC=CC=C65)C)C	528.262

Y2R	70696288	C1CCN(C1)S(=O)(=O)C2=CC=C(C=C2)NC(=S)N3CCC(CC3)C(C4=CC=CC=C4)(C5=CC=CC=C5)O	535.196
Y2R	70685808	CN(C)S(=O)(=O)C1=CC=C(C=C1)NC(=S)N2CCC(CC2)C(C3=CC=CC=C3)(C4=CC=C(C=C4)F)O	527.171

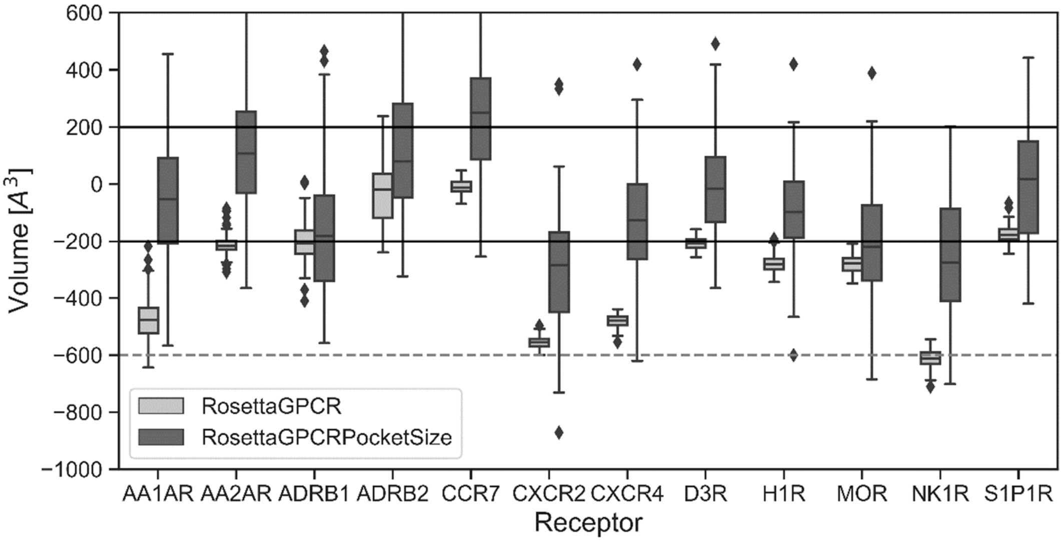

Figure S1. Volume of benchmarked receptors in RosettaGPCR and RosettaGPCRPocketSize. 100 homology models of each receptor were constructed with both modeling approaches and their pocket volume difference to the best determined experimental structure calculated.

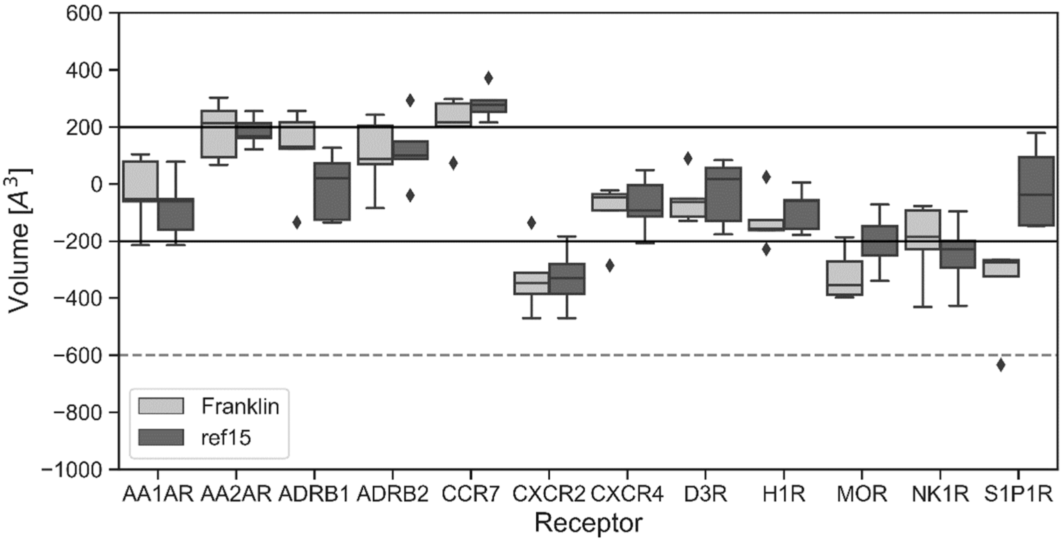

Figure S2. Volume of benchmarked receptors in RosettaGPCRPocketSize and different utilized energy functions for selecting the final best five models are compared. 100 homology models of each receptor were constructed, and their total energy based on different scoring functions determined. The best five models were selected based on the total score of either the Franklin energy function or ref15 energy function.

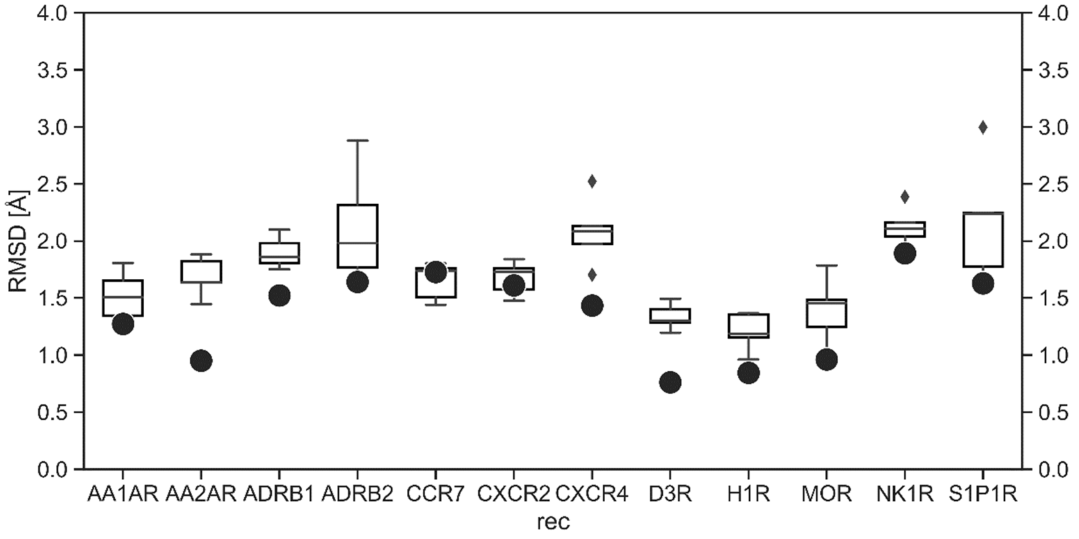

Figure S3. Pocket RMSD of benchmarked receptors in RosettaGPCR and after applying the restraint sets in an additional relaxation step. RMSD between the selected ten residues to calculate the pocket volume as indicator of the pocket RMSD relative to the experimental reference structure for the best model generated with RosettaGPCR (single dot) and the best five models generated with RosettaGPCRPocketSize (box plot) for the GPCRs listed. Only a minor increase of the RMSD with RosettaGPCRPocketSize compared to RosettaGPCR is observed.

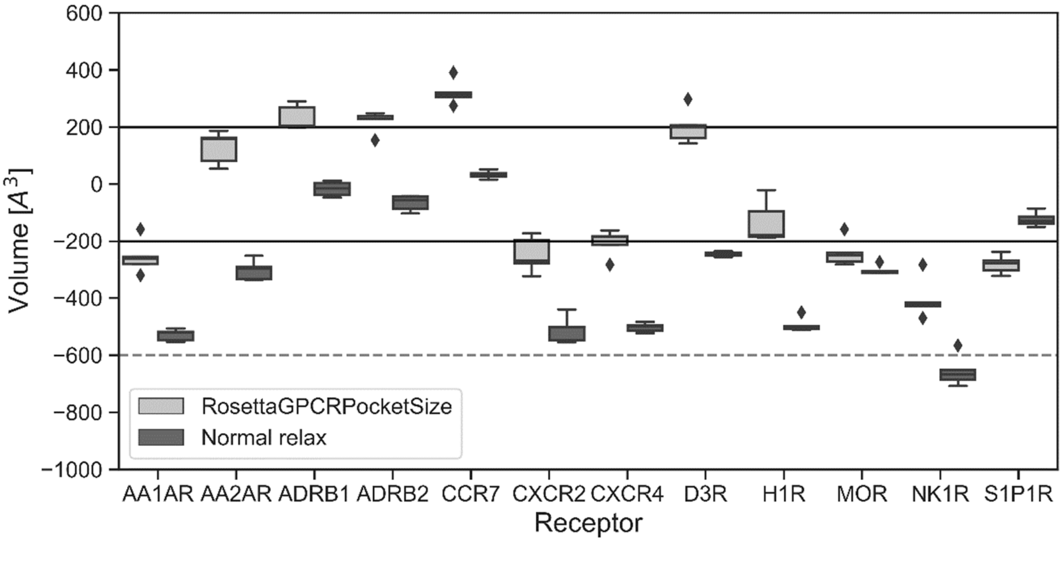

Figure S4. Volume of benchmarked receptors in RosettaGPCR and after applying the restraint sets in an additional relaxation step. The best model generated with RosettaGPCR was repetitively energy-minimized with either the Rosetta relax protocol (Normal relax) or with the RosettaGPCRPocketSize constraint set. From the 100 generated models of each receptor the best five models based on total score for the standard energy-minimization protocol or the best five models based on total score after volume filtering were selected. Pocket volume differences relative to the experimental reference structure are shown. A volume difference below -200Å^3 indicates a pronounced pocket shrinkage, whereas a value above +200Å^3 marks an enlarged pocket volume. Cases with a volume difference smaller or equal to ±200Å^3 fall in the range of the natural pocket size variation which is observed for known GPCR structures. Three times this difference is considered a failed pocket construction.

S1 Protocol. Protocol capture for RosettaGPCRPocketSize. Step-by-step guide to build a pocket ensemble with given scripts in:
https://github.com/FabianLiessmann/RosettaGPCRPocketSize
